# COE Loss-of-Function Analysis Reveals a Genetic Program Underlying Maintenance and Regeneration of the Nervous System in Planarians

**DOI:** 10.1371/journal.pgen.1004746

**Published:** 2014-10-30

**Authors:** Martis W. Cowles, Kerilyn C. Omuro, Brianna N. Stanley, Carlo G. Quintanilla, Ricardo M. Zayas

**Affiliations:** Department of Biology, San Diego State University, San Diego, California, United States of America; University of Oxford, United Kingdom

## Abstract

Members of the COE family of transcription factors are required for central nervous system (CNS) development. However, the function of COE in the post-embryonic CNS remains largely unknown. An excellent model for investigating gene function in the adult CNS is the freshwater planarian. This animal is capable of regenerating neurons from an adult pluripotent stem cell population and regaining normal function. We previously showed that planarian *coe* is expressed in differentiating and mature neurons and that its function is required for proper CNS regeneration. Here, we show that *coe* is essential to maintain nervous system architecture and patterning in intact (uninjured) planarians. We took advantage of the robust phenotype in intact animals to investigate the genetic programs *coe* regulates in the CNS. We compared the transcriptional profiles of control and *coe* RNAi planarians using RNA sequencing and identified approximately 900 differentially expressed genes in *coe* knockdown animals, including 397 downregulated genes that were enriched for nervous system functional annotations. Next, we validated a subset of the downregulated transcripts by analyzing their expression in *coe*-deficient planarians and testing if the mRNAs could be detected in *coe^+^* cells. These experiments revealed novel candidate targets of *coe* in the CNS such as ion channel, neuropeptide, and neurotransmitter genes. Finally, to determine if loss of any of the validated transcripts underscores the *coe* knockdown phenotype, we knocked down their expression by RNAi and uncovered a set of *coe-*regulated genes implicated in CNS regeneration and patterning, including orthologs of *sodium channel alpha-subunit* and *pou4*. Our study broadens the knowledge of gene expression programs regulated by COE that are required for maintenance of neural subtypes and nervous system architecture in adult animals.

## Author Summary

COE transcription factors are conserved across widely divergent animals and are crucial for organismal development. COE genes also play roles in adult animals and have been implicated in central nervous system (CNS) diseases; however, the function of COE in the post-embryonic CNS remains poorly understood. Planarian regeneration provides an excellent model to study the function of transcription factors in cell differentiation and in terminally differentiated cells. In planarians, *coe* is expressed in differentiating and mature neurons, and its function is required for CNS regeneration. In this study, we show that *coe* is required to maintain structure and function of the CNS in uninjured planarians. We took advantage of this phenotype to identify genes regulated by *coe* by comparing global gene expression changes between control and *coe* mRNA-deficient planarians. This approach revealed downregulated genes downstream of *coe* with biological roles in CNS function. Expression analysis of downregulated genes uncovered previously unknown candidate targets of *coe* in the CNS. Furthermore, functional analysis of downstream targets identified *coe*-regulated genes required for CNS regeneration. These results demonstrate that the roles of COE in stem cell specification and neuronal function are active and indispensable during CNS renewal in adult animals.

## Introduction

The Collier/Olfactory-1/Early B-cell factor (COE) family of transcription factors is necessary for animal development. COE proteins possess an atypical HLH domain and a unique zinc finger DNA binding domain conserved across metazoans [1]. Invertebrates encode a single homolog of COE, with roles in mesoderm and ectoderm development [2], [3], whereas vertebrates have four COE paralogs with functions in diverse cell types including B-cells and adipocytes [4]. In the central nervous system (CNS), COE regulates neuronal differentiation, migration, axon guidance, and dendritogenesis during development [2], [3], [5]–[13] and maintains neuronal identity throughout adulthood [14], [15]. COE proteins have also been proposed to function as tumor suppressors [16] and are associated with cancers such as acute lymphoblastic leukemia and glioblastoma [17]–[20]. However, the specific genetic programs regulated by these genes in adult stem cells and mature neurons remain poorly understood.

Stem cells can be studied to determine how transcriptional regulators orchestrate developmental processes or cause disease [21]. An excellent animal model to investigate stem cell regulation *in vivo* is the freshwater planarian *Schmidtea mediterranea* [22]. *S. mediterranea* has the ability to regenerate all tissue types from a population of adult stem cells (called neoblasts). These cells constitute approximately 10–20% of all the cells in the animal and include pluripotent [23] and lineage-committed neoblasts [24]–[29]. The planarian CNS is composed of two cephalic ganglia and a pair of ventral nerve cords that run along the length of the animal, which are comprised of molecularly diverse neuronal subtypes that are regenerated within days after injury or amputation [30]–[32]. Functional analysis of transcription factors in planarians using RNA interference (RNAi) has begun to identify regulatory molecules required for the generation and maintenance of specific neuronal subpopulations in the CNS such as serotonergic and cholinergic neurons [24]–[27], [33]–[35]. Thus, planarians are outstanding organisms to study basic mechanisms that underlie stem cell-based maintenance and regeneration of the adult CNS.

A previous functional screen for transcription factors encoding a helix-loop-helix domain identified a planarian *coe* homolog that is expressed in a small population of neural-committed stem cells (approximately 4–7% of the neoblast pool) and in neurons [24]. We showed that animals fed dsRNA designed to silence *coe* expression (*coe(RNAi)* animals) regenerated abnormal brains; furthermore, uninjured *coe(RNAi)* planarians displayed behavioral defects and reduced expression of neural subtype-specific genes [24]. In this study, we sought to identify genes regulated by *coe* with roles in CNS renewal by comparing the transcriptome profiles of uninjured control and *coe(RNAi)* animals, uncovering differentially expressed genes with predicted roles in CNS function. We validated a subset of these genes by testing for loss of expression after *coe* knockdown and visualizing their expression in *coe^+^* cells. These analyses revealed a set of nine candidate targets of *coe* in adult neurons, many of which are important for neuronal subtype identity (e.g., ion channels, neuropeptides, and neurotransmitters). In addition, our findings demonstrate that *coe* functions to drive gene expression in multiple neuronal classes, including excitatory and inhibitory neurons. To gain insights into the roles candidate COE targets play in CNS turnover and repair, we analyzed the function of downregulated transcripts using RNAi. Our functional screen identified several genes required for CNS regeneration, including homologs of a voltage-gated sodium channel α-subunit (*scna-2*) and the transcription factor *pou4l-1*. Our results suggest that COE is required for the expression of neural-specific genes in differentiating and mature neurons, a function that is essential to maintain CNS architecture and regulate neuronal regeneration.

## Results/Discussion

### *coe* is required for maintenance of nervous system structure

**Figure 1:**
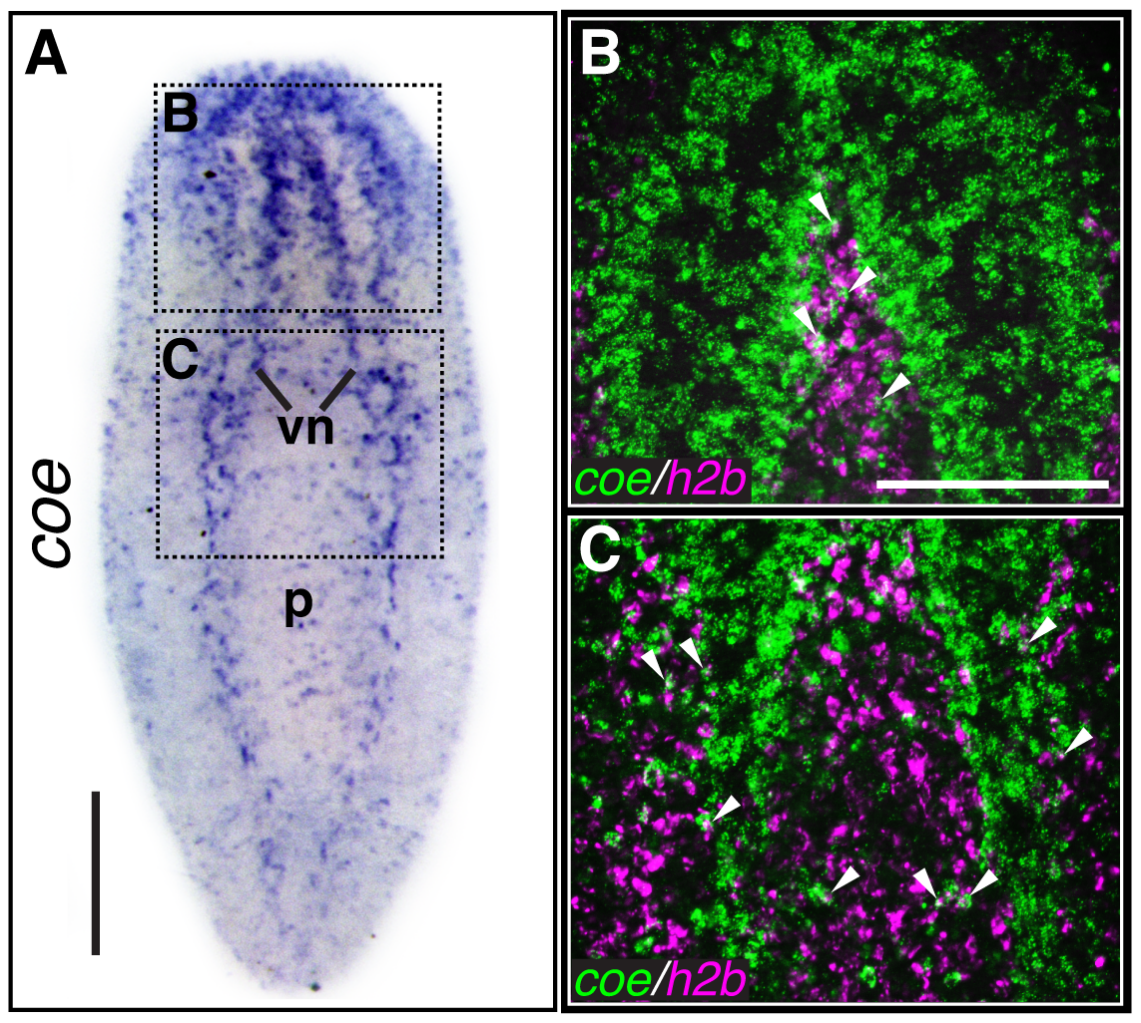
*coe* is expressed in the nervous system and a subset of cycling stem cells. (**A**) *In situ* hybridization to *coe* in *S. mediterranea* (vn, ventral nerve cords; p, pharynx). Dashed boxes show regions imaged in B–C (N≥10). (**B–C**) Double-fluorescent *in situ* hybridization to *coe* and *h2b*. Arrowheads mark examples of double-labeled cells (N = 14). Anterior is up in all panels. Scale bars, A = 200 µm, B = 100 µm.

Using an optimized whole-mount *in situ* hybridization protocol (WISH) (see Materials and Methods), we found that *coe* mRNA was primarily restricted to neurons in *S. mediterranea* (Fig. 1A). In agreement with our previous findings [24], we also observed *coe* transcripts in a subset of cycling stem cells (*h2b* ^+^) (Fig. 1B–C). We previously reported that *coe(RNAi)* animals regenerate cephalic ganglia that fail to connect at the anterior commissure and have significantly smaller brains with fewer *cpp-1^+^*, *npp-4^+^*, and *npy-2^+^* neurons when compared to the controls [24]. This defect is not restricted to the anterior portion of the animal. Additional experiments showed *coe(RNAi)* animals do not properly regenerate their ventral nerve cords (Fig. S1A–B). Moreover, analysis of the brain patterning defect using anti-VC-1, a marker of the photoreceptor neurons and their axons, revealed that the optic chiasm failed to connect at the midline in *coe(RNAi)* animals (Fig. S1C). These data demonstrate that *coe* is essential for neuronal regeneration at both anterior and posterior facing wounds and that *coe* regulates genes required for reestablishing midline patterning following brain amputation.

**Figure 2:**
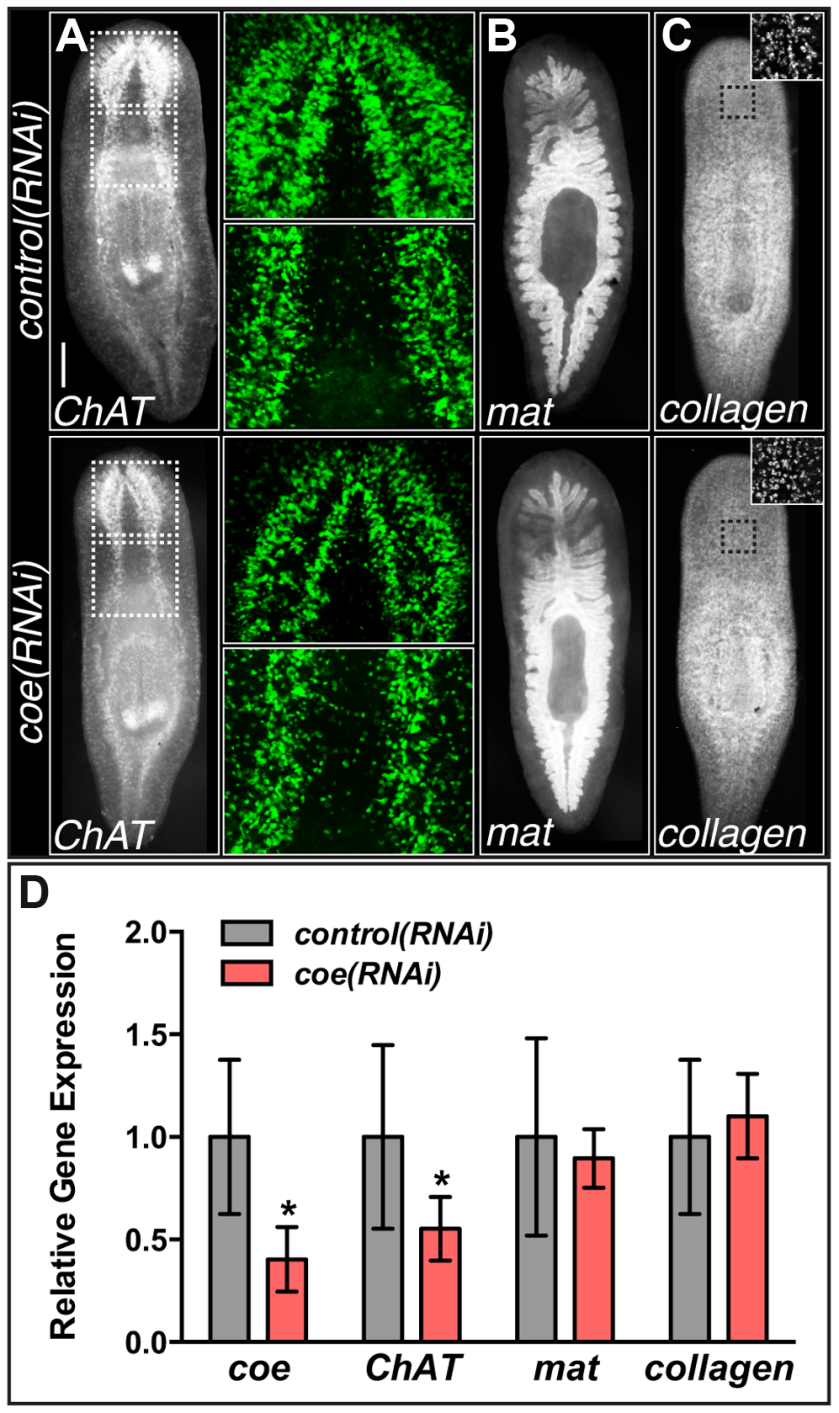
*coe* RNAi strongly inhibits the expression of *ChAT* in intact planarians. (**A–C**) *coe* RNAi-treated animals were processed for fluorescent *in situ* hybridization (FISH) to *ChAT* (N = 10 for each treatment), *mat* (N = 3 control and 4 RNAi planarians), or *collagen* (N = 7 control and 5 RNAi). White dashed boxes in A denote regions imaged at higher magnification shown in the panels to the right. Black dashed boxes in C denote regions imaged at higher magnification shown in top right insets. (**D**) RT-qPCR experiments measuring the relative expression of *coe*, *ChAT*, *mat*, or *collagen* in *control(RNAi)* or *coe(RNAi)* planarians following the 6^th^ RNAi treatment. Graph shows the mean ± s.d. expression levels relative to the controls. *P<0.05, Student's t-test.

In addition, we previously noted that silencing of *coe* in intact uninjured animals results in a reduction of *ChAT^+^* and *pc2^+^* neurons near the anterior commissure and a loss of *cpp-1^+^* neurons. Following the 6^th^ feeding of *coe* dsRNA, 100% of the animals exhibited impaired negative phototaxis [24]. To investigate the specificity of the *coe* knockdown phenotype on the CNS, we examined the effect of *coe* RNAi on the intestine and muscle as representative endodermal or mesodermal tissues, respectively. We hybridized uninjured control and *coe(RNAi)* animals with riboprobes specific to *ChAT* (as a positive control), *mat* [36], and *collagen* [37]. As expected, we observed a decrease in *ChAT* ^+^ neurons in the head [24] and noted a decrease in *ChAT* expression throughout the animal (Fig. 2A); by contrast, we did not observe a change in the spatial distribution of *mat* or *collagen* following *coe* knockdown (Fig. 2B–C). To quantify the effect of *coe* RNAi treatments on the expression of *ChAT*, *mat* and *collagen*, we measured relative mRNA levels by reverse transcription quantitative PCR (RT-qPCR). First, we confirmed *coe* knockdown led to a significant decrease in the relative expression of *coe* mRNA (down 60%±16% compared to the controls; Fig. 2D). Measurement of *ChAT*, *mat* and *collagen* from *coe(RNAi)* planarians revealed that *ChAT* mRNA levels were significantly down (45%±15%) compared to control animals; in contrast to *ChAT*, the relative mRNA levels of *mat* or *collagen* were not affected by *coe* RNAi treatment (Fig. 2D). Combined with our previous work [24], these results strongly suggest that *coe* knockdown specifically affects gene transcription in the nervous system and does not cause obvious defects in other tissues such as the intestine or muscle. Furthermore, our results are consistent with reports demonstrating that COE is required to maintain cholinergic and peptidergic neuronal subtype-specific gene expression in *Caenorhabditis elegans* and *Drosophila melanogaster* [14], [15].

**Figure 3:**
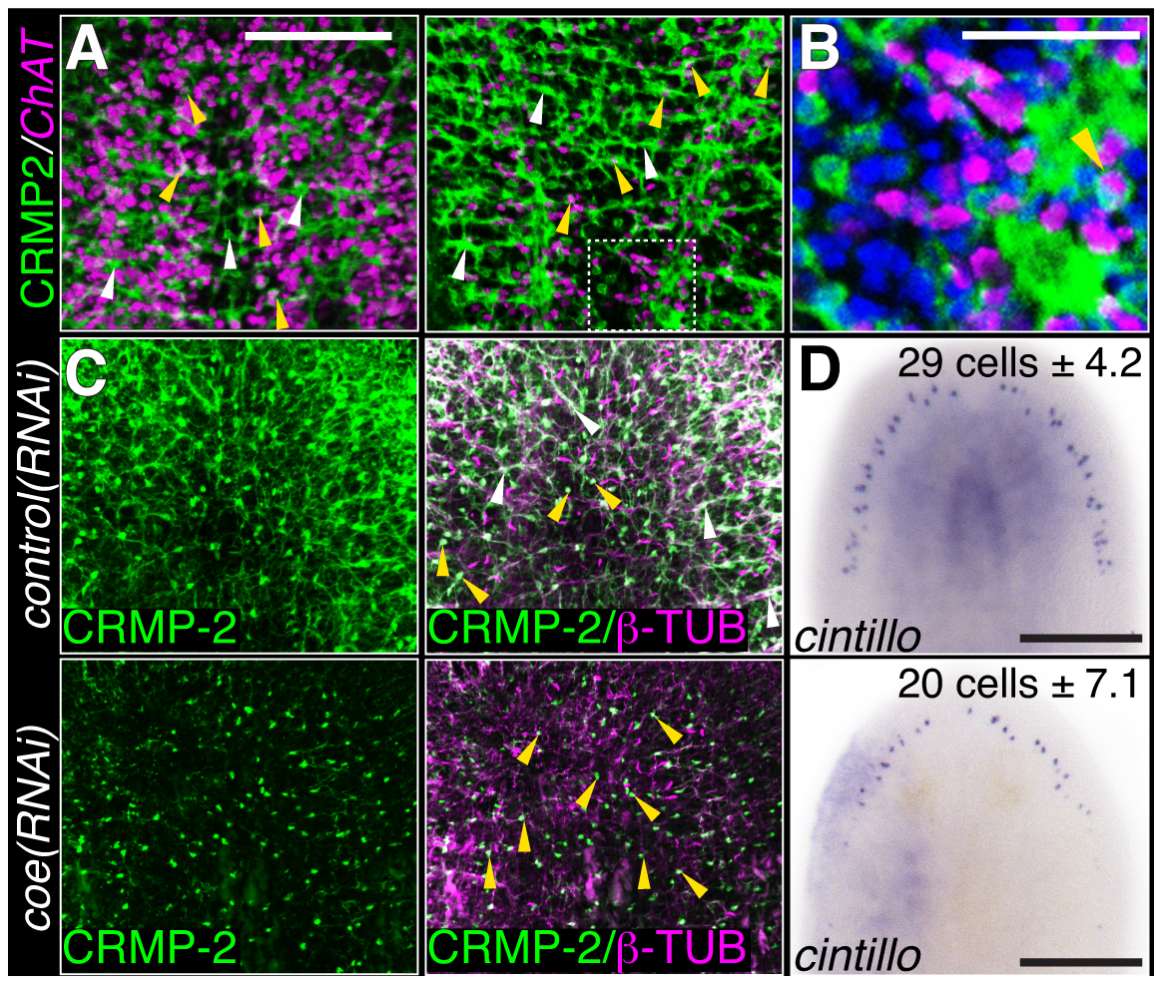
COE function is required for maintenance of nervous system architecture in uninjured planarians. (**A**) Head or tail images from an animal stained with anti-CRMP-2 and processed for FISH to *ChAT*. CRMP-2 is expressed in axon projections (white arrows) and neuronal cell bodies (yellow arrows; N = 7). (**B**) Higher magnification image of region denoted by white box in D shows CRMP-2 is detected in *ChAT* ^+^ cell bodies (arrowhead). Nuclei were stained with DAPI (blue). (**C–D**) Uninjured control and *coe(RNAi)* planarians labeled with anti-CRMP-2 and anti-β-TUBULIN or processed for *in situ* hybridization to *cintillo*. White and yellow arrows point to axon projections and cell bodies, respectively. N = 8 animals for each treatment; 412 and 290 *cintillo^+^* cells were counted from control and *coe(RNAi)* animals, respectively. The number in the top right corner indicates the mean ± s.d. of *cintillo^+^* cells; *P<0.05, Student's t-test. Anterior is up in all panels. Scale bars, A = 200 µm, D = 100 µm, E = 50 µm, and G = 200 µm.

To investigate if the inhibition of *coe* perturbs nervous system architecture downstream of gene expression changes, we labeled neuronal cell bodies and their projections using anti-CRMP-2, which labels a subset of neuronal cell bodies and their axon projections, and anti-β-tubulin to visualize nerve projections (Fig. 3A–C). In *coe(RNAi)* animals, we observed a striking decrease in axon projections labeled by anti-CRMP-2 and anti-β-tubulin compared to the controls; however, expression of CRMP-2 was retained in the cell bodies (Fig. 3C). In addition, when we labeled sensory neurons using *cintillo* [38], *coe(RNAi)* animals exhibited significantly fewer *cintillo^+^* cells (Fig. 3D). Our results strongly suggest that nervous system architecture is severely reduced or lost in the absence of *coe*. These structural defects likely underlie the behavioral abnormalities observed in *coe-*deficient planarians.

### Identification of genes regulated by *coe* in the planarian nervous system

Although COE has been shown to drive differentiation of several classes of neurons during development [39], the transcriptional programs controlled by this transcription factor in adult nervous system function are poorly defined. We reasoned that the CNS-specific *coe* RNAi phenotype in intact planarians represents an excellent opportunity to identify gene expression programs controlled by COE in the post-embryonic nervous system. Thus, we used comparative mRNA sequencing (RNA-seq; see Materials and Methods) to sequence mRNAs isolated from uninjured controls and *coe(RNAi)* animals one week after the 6^th^ RNAi treatment, which was the point in time we consistently observed behavioral defects and loss of neural-specific gene expression in 100% of *coe-*deficient animals and did not detect overt defects in other tissues (Fig. 2). RNA-seq analysis identified 909 differentially expressed genes; 397 were downregulated, and 512 were upregulated (Table S1). Functional annotation using DAVID software showed that the set of downregulated genes was significantly enriched for Gene Ontology (GO) terms associated with “ion channel,” “neuronal activities,” “nerve-nerve synaptic transmission,” “voltage-gated ion channel,” and “cell adhesion molecule”; by contrast, the upregulated genes were enriched for GO terms associated with “cytoskeletal protein” and “muscle development” (Table 1). *coe* mRNAs were not detected in a muscle pattern (Fig. 1), nor did we detect overt phenotypes associated with muscle differentiation (Fig. 2). However, the RNA-seq data raised the possibility that *coe* might negatively regulate mesoderm specification, which is required for muscle development [3], [40]. It is possible upregulation of muscle genes is an indirect consequence of a loss of nervous system influence such as cholinergic transmission and/or neuropeptide regulation. Previous studies have demonstrated cholinergic neurotransmission is required for coordinated muscle contractions in planarians [41]–[43]. Thus, we speculate that loss of nervous system modulation disrupts muscle homeostasis and leads to changes in expression of muscle-related genes. Although our experiments do not definitively assign the role of COE in muscle differentiation or maintenance, our data do clearly indicate that *coe* is required for expression of nervous system-specific genes in adult planarians.

**Table 1:** Annotation of genes differentially expressed in *coe(RNAi)* animals using DAVID software.

Functional Cluster	Enrichment Score	Differential Expression
Ion channel	7.65	Downregulated
Neuronal activities	6.50	Downregulated
Voltage-gated ion channel	2.38	Downregulated
Microtubule binding motor protein	2.31	Downregulated
Nerve-nerve synaptic transmission	1.71	Downregulated
Cell adhesion molecule	1.30	Downregulated
Neurogenesis	1.27	Downregulated
Muscle contraction	4.13	Upregulated
Cytoskeletal protein	3.98	Upregulated
Mitosis	2.25	Upregulated

**Figure 4:**
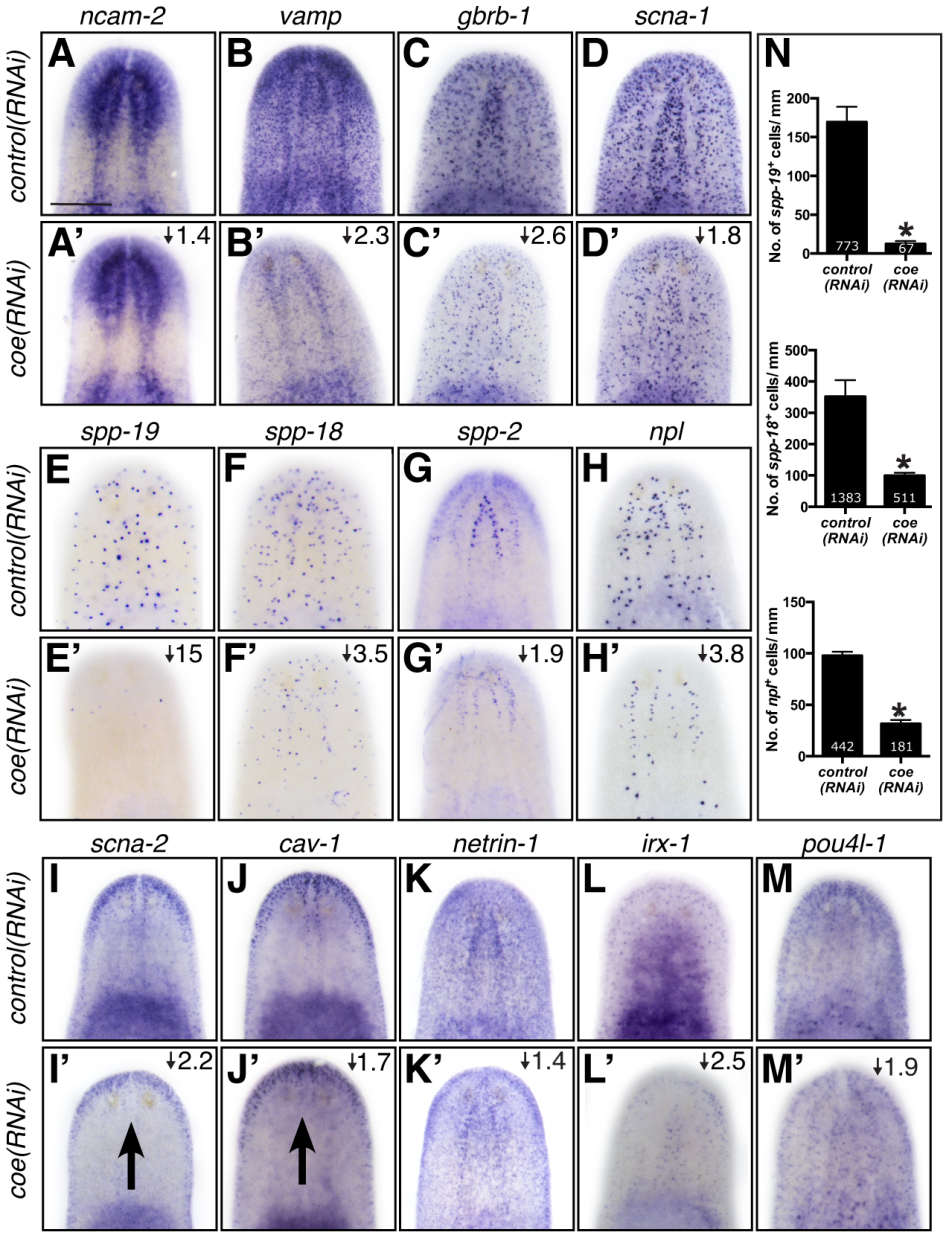
The expression pattern of nervous system genes downstream of COE is changed or severely reduced following *coe* RNAi. Control and *coe(RNAi)* treated animals were processed for *in situ* hybridization to the genes indicated above each panel (N≥5 animals per treatment). (**A–M′**) Detection of all genes was reduced following *coe* knockdown. Numbers in top right corner represent linear fold changes in mRNA expression in *coe(RNAi)* planarians relative to the controls. Arrows in I′ and J′ point to loss of expression at the midline compared to the controls (I and J). (**N**) Quantification of *spp19^+^*, *spp-18^+^*, and *npl* ^+^ cells (N = 3); the total number of cells counted is indicated within each bar. Error bars in all graphs are s.d. from the mean; *P<0.05, Student's t-test. Anterior is up in A–M′. Scale bar in A = 100 µm.

Based on the annotation of differentially expressed genes, we hypothesized that genes predicted to play roles in nervous system functions in the downregulated category likely include direct COE targets. To test our hypothesis and validate genes found in our RNA-seq dataset, we selected 65 genes that were dramatically downregulated, associated with neural functions, or annotated as transcription factor homologs. First, we performed WISH to determine the tissue-specific pattern of expression of all 65 genes (representative examples are shown in Fig. 4). As we expected, the most prominent mRNA expression pattern was in the nervous system (26 of 65 genes; see Table S2), similar to *ChAT* and *cpp-1*, which we had previously found to be putative downstream targets of COE [24]. In addition, we observed genes that were expressed broadly in the nervous system (such as *neural cell adhesion molecule-2* (*ncam-2*), *vesicle-associated membrane protein like-1* (*vamp*), *gamma-aminobutyric acid receptor subunit beta like-1* (*gbrb-1*), and *voltage-gated sodium channel alpha-1* (*scna-1*)) or in discrete neuronal subpopulations (such as *secreted peptide prohormone-19*, *-18*, *-2* (*spp-19*, *-18*, *-2*), *neuropeptide like* (*npl*), *voltage-gated sodium channel alpha-2* (*scna-2*), and *caveolin-1* (*cav-1*)) (Fig. 4A–J). Our list also included transcripts that labeled subsets of neurons in the brain (such as *netrin-1*) (Fig. 4K) [44]. In addition, we found that the transcription factors *iroquios-1* (*irx-1*) and *pou class 4 transcription factor 4 like-1* (*pou4l-1*) were expressed at or near the cephalic ganglia (Fig. 4L–M), and their mRNA was detected in *ChAT* ^+^ neurons by fluorescent *in situ* hybridization (FISH) (Fig. S2). Next, we tested the effect of *coe* RNAi on the expression of 33 genes that could be visualized in discrete cell populations by WISH. Knockdown of *coe* led to a marked reduction in the expression of 31 genes (Table S2; representative results are shown in Fig. 4A′–H′, K′–M′); for two genes, *scna-2* and *cav-1*, we observed a loss of expression at the midline (Fig. 4I′–J′). Furthermore, we quantified the number of cells labeled by *spp-19*, *spp-18*, and *npl* probes. As expected, we found there was a significant reduction in the number of *spp-19^+^*, *spp-18^+^*, and *npl^+^* cells following *coe* RNAi (Fig. 4N).

**Figure 5:**
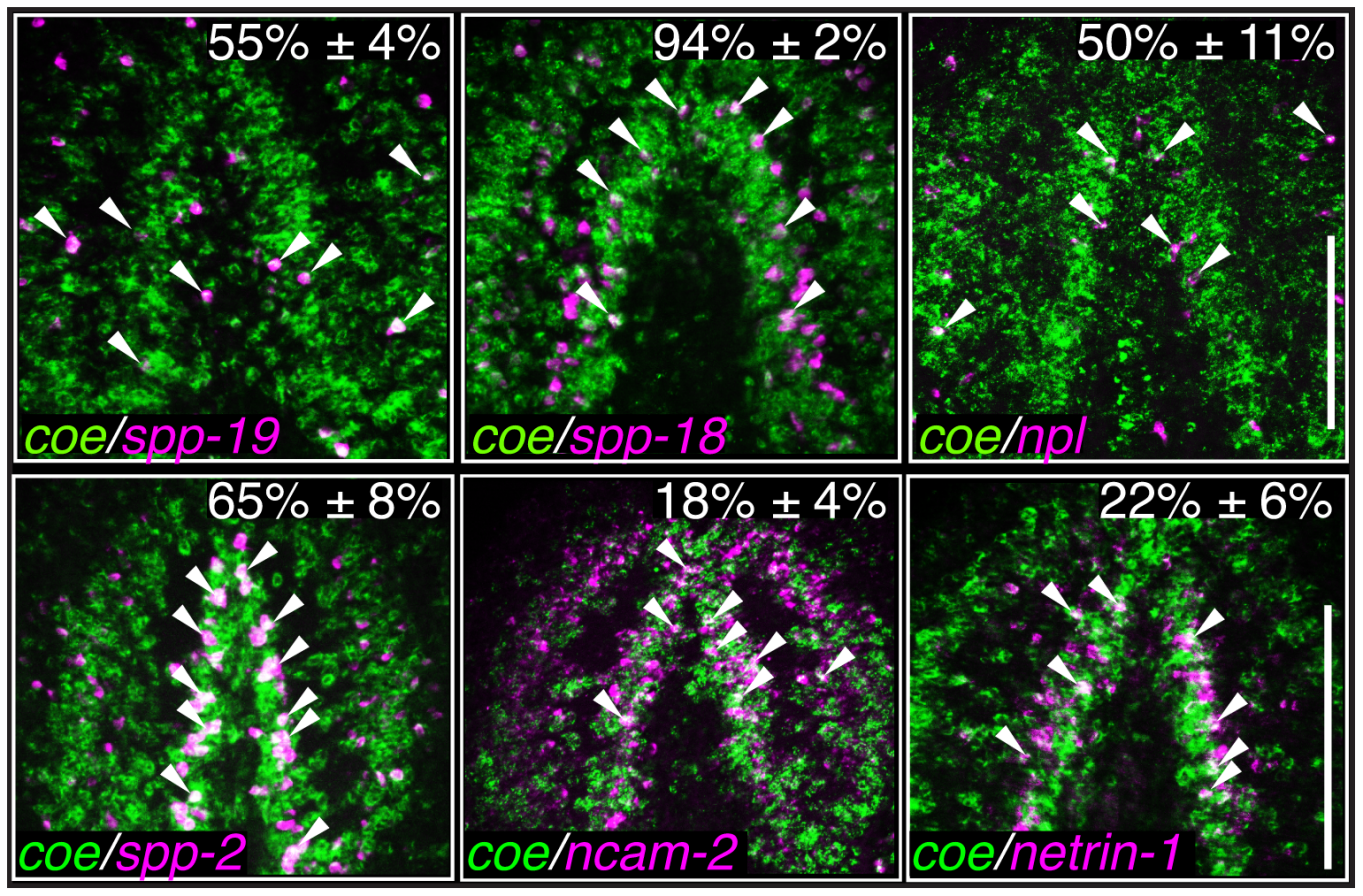
Identification of genes expressed *coe^+^* neurons. Fluorescent *in situ* hybridization to *coe* and either *spp-19*, *spp18*, *npl*, *spp-2*, *ncam-2*, or *netrin-1*. Percentages indicate the proportion ± s.d. of cells that were also *coe^+^* (N = 110 *spp-19^+^*, 319 *spp-18^+^*, 173 *npl^+^*, 202 *spp-2*, 236 *ncam-2*, and 141 *netrin-1* cells counted from 2–3 animals per group). Arrowheads mark double-labeled cells. Anterior is up in all panels. Scale bars = 100 µm.

As an additional test to validate the *in situ* hybridization results, we measured the relative expression levels of downregulated genes in control and *coe* RNAi-treated planarians using RT-qPCR (Fig. S3A). All of the genes we tested showed a decrease in relative expression following *coe* RNAi (9 of 14 genes were significantly downregulated; P<0.05, Student's t-test). By contrast, when we measured the relative expression of CNS-expressed genes that were not on our list of differentially expressed genes, none were significantly reduced (11 of 11 genes; Fig. S3B–C). Although some of the control genes we selected were reduced near levels comparable to some genes downregulated following *coe* RNAi (e.g., *ncam2*, *vamp*, and *gbrb1*; Fig. S3A), we noted that *isotig13897* and *npp-2* [30], which are transcripts detected in subsets of neurons or throughout the CNS, respectively, remained unchanged (Fig. S3B–C). It is possible that some changes in gene expression associated with *coe* RNAi are consequence of a reduction in nervous system tissue. We proceeded to perform double-FISH to *coe* and validated genes to determine if any were potential genetic targets of COE. Of the 17 genes we were able to reliably detect by FISH (33 genes were tested; see Table S2), 11 were expressed in *coe^+^* cells (representative results are shown in Fig. 5 and Fig. S4), including *ChAT* and *cpp-1* [24]. Together, these results identified nine novel candidate targets of COE in the nervous system, including genes important for maintaining neuronal subtype identity such as ion channels, ion channel receptors, and neuropeptide genes (Table 2). In addition, our data suggest that COE is essential to maintain genetic programs in multiple classes of adult neuronal subtypes including excitatory (cholinergic) and inhibitory (GABAergic) neurons.

**Table 2:** Candidate COE targets genes identified in *S. mediterranea*.

Gene Name	CNS function
*Smed-gamma-aminobutyric acid receptor subunit gamma like (gbrg)*	Neurotransmitter receptor
*Smed-netrin-1*	Axon Guidance
*Smed-neural cell adhesion molecule-2 (ncam-2)*	Cell adhesion
*Smed-neuropeptide like-1 (npl-1)*	Novel gene; Unknown
*Smed-secreted peptide prohormone 18 (spp-18)*	Neuropeptide
*Smed-secreted peptide prohormone 19 (spp-19)*	Neuropeptide
*Smed-secreted peptide prohormone-2 (spp-2)*	Neuropeptide
*Smed-vesicle-associated membrane protein like-1 (vamp)*	Cell adhesion
*Smed-voltage-gated sodium channel (scna-1)*	Ion Channel

### Genes downstream of *coe* are required for proper CNS regeneration

**Figure 6:**
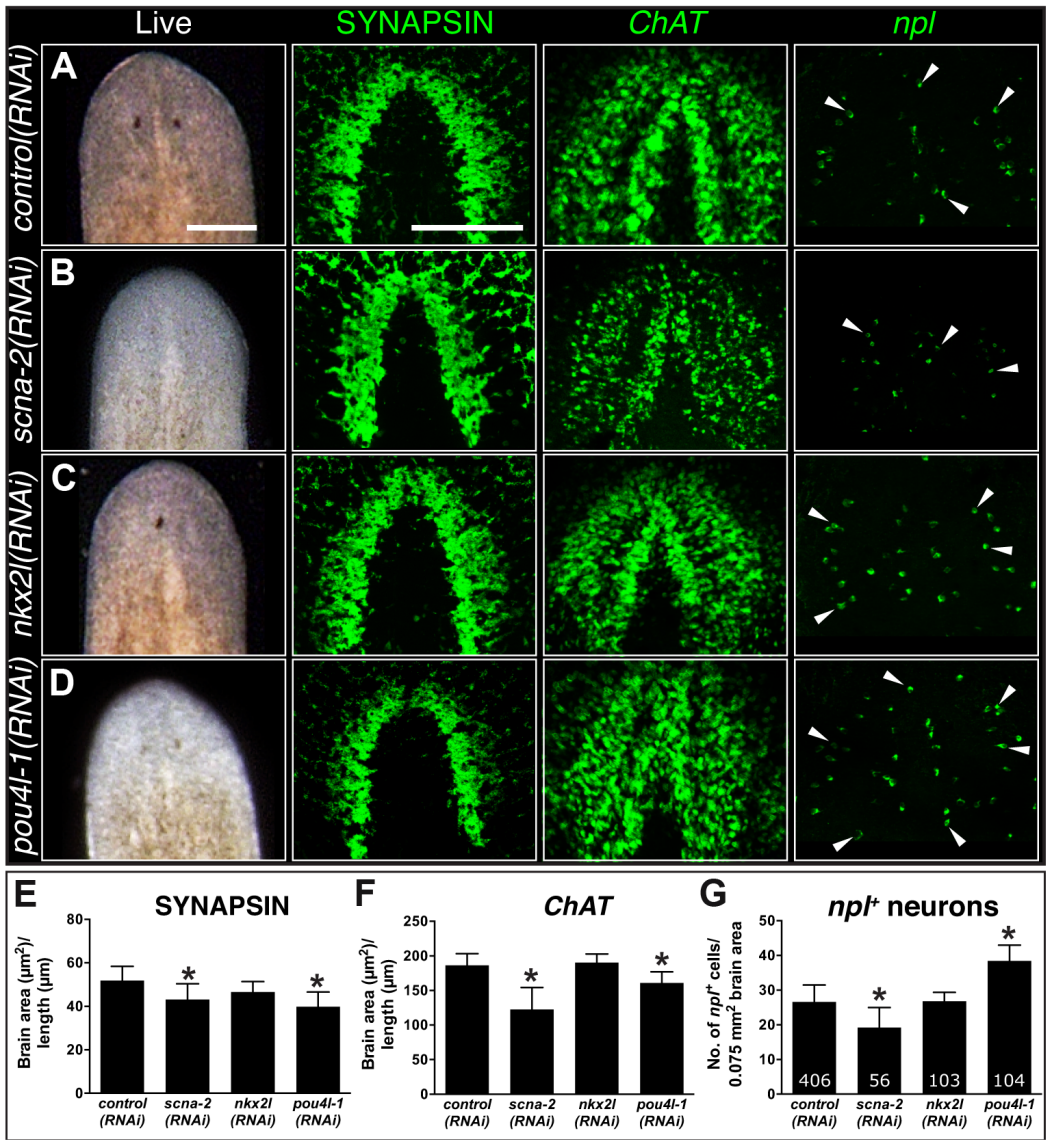
CNS regeneration defects following knockdown of COE-regulated genes. (**A–D**) Animals were fed control, *scna-2*, *nkx2l* and *pou4l-1* bacterially-expressed dsRNA (indicated to the left of each panel), amputated pre-pharyngeally and allowed to regenerate. Ten-day regenerates were imaged live (A–D), killed and immunostained with anti-SYNAPSIN or processed for fluorescent *in situ* hybridization to *ChAT* or *npl* (N≥4). (**E–F**) Brain size estimated by measuring head area stained by anti-SYNAPSIN or *in situ* hybridization to *ChAT* and normalized by the length of animal for *control*, *scna-2*, *nkx2l*, *and pou4l-1* RNAi planarians. (**G**) Quantification of *npl^+^* cells normalized by brain size measured from *ChAT* stain in F (N≥4 animals in each group); the total number of *npl^+^* cells counted is indicated within each bar. Error bars in all graphs are s.d. from the mean; *P<0.05, Student's t-test. Anterior is up in A–D. Scale bars = 100 µm.

Our RNA-seq dataset revealed that *coe* is essential to maintain the expression of hundreds of genes in the adult animal. This change in the neuronal gene expression landscape led to abnormal CNS structure and behavior. To identify genes downstream of *coe* that contribute to CNS differentiation, we took advantage of the experimental ease in examination of gene function in planarian regeneration and analyzed the role of 11 downregulated genes that were expressed in neurons or predicted to encode transcription factors (Table 3). Following RNAi, animals were amputated pre- and post-pharyngeally and allowed to regenerate for 10 days. We found that 6 out of 11 genes resulted in defective brain regeneration (see Table 3); *scna-2*, *pou4l-1*, and *nkx2l* caused the strongest phenotypes. Compared to the controls, *scna-2(RNAi)* animals had less eye pigmentation or developed a single eyespot; *nkx2l(RNAi)* animals exhibited photoreceptor defects; and *pou4l-1(RNAi)* animals had less photoreceptor pigment (Fig. 6A–D). To examine CNS architecture, we stained *scna-2*, *nkx2l*, and *pou4l-1* RNAi treated planarians with anti-SYNAPSIN and the *coe-*regulated genes *ChAT* and *npl*. Although subtle, all three showed abnormalities in brain morphology (Fig. 6A–D). However, when we measured the area of the brain stained by anti-SYNAPSIN, only *scna-2* and *pou4l-1* RNAi animals had a significant reduction in neuropil density (Fig. 6E). Consistent with this observation, the *ChAT^+^* brain areas were smaller in *scna-2(RNAi)* and *pou4l-1(RNAi)* animals (Fig. 6F) but not in *nkx2l(RNAi)* animals. The smaller brain phenotype was accompanied by fewer *npl^+^* neurons in *scna-2(RNAi)* animals; however, despite their smaller brains, *pou4l-1(RNAi)* animals regenerated significantly more *npl^+^* cells than controls (Fig. 6G). These findings demonstrate that *scna-2* is required for CNS regeneration and highlight the importance of ion channels in neurogenesis regulation during CNS development, maintenance, and repair [45]–[47]. Interestingly, these data suggest that *pou4l-1* plays a role in the specification of certain neuronal lineages. It is possible that in the absence of *pou4l-1*, planarians regenerate the incorrect proportion of neuronal subtypes and have disorganized brains, but this possibility will require further analysis with additional neuronal subtype-specific markers. By contrast, our results suggest *nkx2l* is not required for CNS regeneration *per se*. Following *coe* RNAi, *nkx2l* expression was reduced by *in situ* hybridization and RT-qPCR (Table S2 and Fig. S3A), but *nkx2l*, which is primarily expressed in stem cells and in progeny [48], was not detected in the nervous system (Fig. S5A). We hypothesize *nkx2l* functions in early regeneration to establish patterning, which is consistent with the observation that *nkx2l(RNAi)* planarians fail to regenerate properly patterned head (Fig. 6C) and tail tissues (Fig. S5B).

**Table 3:** Functional analysis of genes downregulated following *coe* RNAi.

Gene Name	RNAi Phenotype	DAVID Annotation
*Smed-gamma-aminobutyric acid receptor subunit beta like (gbrb1)*	No phenotype observed (12/15)	N-N synaptic transmission, Ion channel, Neural activities
*Smed-gamma-aminobutyric acid receptor subunit gamma like (gbrg)*	No external phenotype observed (34/35), reduced neuropil density at anterior commissure (6/13)	N-N synaptic transmission, Ion channel, Neural activities
*Smed-hemicentin-1*	No phenotype observed (13/14)	Cell Adhesion
*Smed-iroquois-1 (irx-1)*	No phenotype observed (10/10)	Transcription Factor
*Smed-nkx2 like-1 (nkx2l-1)*	Photoreceptor defects (9/35), abnormal brain architecture (35/35), and reduced or indented tail blastemas (7/35)	Transcription Factor
*Smed-notch-1*	No phenotype observed (10/10)	Neurogenesis
*Smed-neuropeptide like (npl)*	Delayed photoreceptor regeneration (9/33)	NA
*Smed-pou class 4 transcription factor 3 like-1 (pou4l-1)*	Lighter photoreceptors (5/40) and reduced neuropil density (14/20)	Transcription Factor
*Smed-voltage-gated sodium channel (scna-1)*	No phenotype observed (15/15)	Voltage-gated ion channel, Ion channel, Neural activities
*Smed-voltage-gated sodium channel (scna-2)*	Reduced photoreceptor formation (20/41) and neuropil density (10/10); regenerated a single photoreceptor (1/41)	Voltage-gated ion channel, Ion channel, Neural activities
*Smed-voltage-gated sodium channel (scna-3)*	Delayed photoreceptor formation (6/28), reduced neuropil density at anterior commissure (6/14)	Voltage-gated ion channel, Ion channel, Neural activities

The number of animals showing the phenotype(s) among the total number examined from at least two independent experiments is indicated in parentheses.

It is noteworthy that several transcription factors that we identified in our screen are putative COE targets in *Xenopus* development, including *irx-1*, *tal*, *pou4l-1*, and *nkx2l* [39]. Of these genes, we found that expression of *pou4l-1* was important for CNS regeneration and *nkx2l* was involved in patterning. NKX and POU orthologs play critical roles during CNS development of invertebrate and vertebrate organisms [49]–[51]. These data suggest that regulatory genes downstream of COE are conserved and have roles in CNS regeneration. However, it will be important to experimentally resolve whether these transcription factors are *bona fide* targets of COE in planarians or other animals such as *Xenopus*.

### Concluding remarks

**Figure 7:**
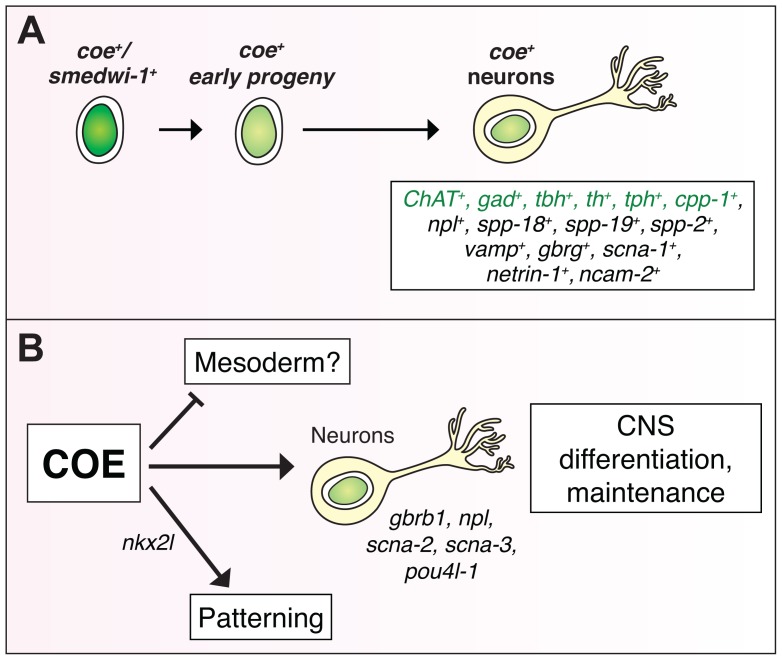
COE function is required for differentiation and maintenance of diverse neuron types. (**A**) *coe* is expressed in lineage-committed neoblasts (*smedwi^+^*) and early progeny [24], and diverse neuron types, including cholinergic (*ChAT*), GABAergic (*gad*), octopaminergic (*tbh*), dopaminergic (*th*), serotonergic (*tph*), and neuropeptidergic (*cpp-1*, *npl*, *spp-18*, *spp-19*, *spp-2*) neurons. Genes in green were identified in [24]. (**B**) To gain insights into how loss of COE function contributes to defects in nervous system differentiation, we analyzed the function of genes that were downregulated in *coe(RNAi)* animals. These analyses identified additional genes required for CNS regeneration (*gbrb1*, *npl*, *scna-2*, *scna-3*, *pou4l-1*) and patterning (*nkx2l*). In *coe(RNAi)* animals, we also detected upregulated genes enriched for GO terms associated with muscle development (Table 1), suggesting that COE may also function to repress the expression of mesoderm-specific genes.

COE proteins are known to function as terminal selectors of neuronal identity in adult organisms [14], [15], [52], yet the neuronal subtypes and specific genetic programs regulated by COE in the adult CNS are not well understood. In this study, we exploited the high rate of tissue turnover and regenerative capacity of planarians to expand our understanding of how COE may function in the post-embryonic nervous system. We combined RNAi with RNA-seq analysis and identified a set of differentially expressed genes associated with nervous system biological roles. Expression analysis of a subset of these genes revealed novel candidate targets of *coe* in planarian neurons (Fig. 7A), some of which underscored *coe*'s essential role in maintaining expression of genes vital for neuronal subtype identity and function (such as neurotransmitter receptors, ion channels, and neuropeptide encoding genes) (Fig. 7A–B). Decoding which transcriptional changes are direct or indirect consequences of *coe* loss in the planarian model will be vital to further elucidate how mutations in COE proteins cause or contribute to disease pathologies in the CNS. The next step will be to find direct COE binding sites genome-wide using *in silico* and chromatin immunoprecipitation (ChIP) approaches and combining these findings with our differential expression data. In addition, molecular profiling of *coe^+^* cell populations (such as stem cells, postmitotic progeny, and neurons) will be essential to determine how *coe* function alters in cell type-specific contexts. In conclusion, our study demonstrates the importance of COE family proteins in neuronal turnover and repair of the adult CNS and broadens our understanding of the regulatory programs governed by these factors.

## Materials and Methods

### Animal husbandry

Asexual *Schmidtea mediterranea* (CIW4) were reared in 1× Instant Ocean Salts (0.83 mM MgSO_4_, 0.9 mM CaCl_2_, 0.04 mM KHCO_3_, 0.9 mM NaHCO_3_, and 0.21 g/L Instant Ocean Aquarium Salt diluted in ultra-pure water) at 20°C. Animals were starved for one week, and those ranging between 2–5 mm in length were used for experimentation.

### RNA interference

Animals were administered six feedings of bacterially expressed dsRNA complementary to the indicated gene over three weeks as previously described [53]; *gfp* dsRNA was fed as a control. Unless otherwise indicated, all intact RNAi animals were fixed seven days following the 6^th^ dsRNA treatment. For regeneration experiments, planarians were amputated pre- and post-pharyngeally 24 hours following the 6^th^ dsRNA feeding.

### Whole-mount *in situ* hybridizations and immunostainings

Animals were processed for colorimetric whole-mount *in situ* hybridization using the protocol described in [54]. Fluorescent *in situ* hybridization experiments were performed as described in [24], [54] and developed using Tyramide Signal Amplification (TSA) as described in [55]. Briefly, animals were incubated for 5 min. in borate buffer (100 mM borate pH 8.5, 0.1% Tween-20) and then developed in TSA Reaction Buffer (borate buffer, 2% dextran sulfate, 0.1% Tween-20, 0.003% H_2_O_2_), containing fluor-tyramide and 4-iodophenylboronic acid for 30 min. For double-FISH, animals were quenched in 1% H_2_O_2_ for 1 hour. For γ-irradiation experiments, animals were fixed 6 days following a 100 Gy treatment, a time point when both stem cells and postmitotic progenitors are ablated. Accession numbers for the sequences used in this study are listed in Table S3. For immunostaining with anti-SYNORF1 (1∶400, 3C11, DSHB) or anti-VC-1 (1∶10,000; kindly provided by Hidefumi Orii), animals were fixed with Carnoy's solution [56]. For anti-CRMP-2 (1∶50, 9393S, Cell Signaling) or anti-β-TUBULIN (1∶1000; E7, DSHB) labeling, animals were fixed with formaldehyde, processed without a reduction step, and labeled using TSA [54].

### RNA sequencing and DAVID analysis

One week after the final dsRNA treatment, RNA was extracted from three independent control and *coe(RNAi)* animal groups using Trizol (Life Technologies). RNA samples were treated with DNase using the Turbo DNA-free Kit (Life Technologies) and purified using the RNeasy MinElute Cleanup kit (Qiagen). Sequencing libraries were synthesized using the TruSeq RNA Sample Prep Kit v2 and sequenced on a HiSeq 2000 System (Illumina). More than 12 million 100-bp single-end reads were generated for each sample. Sequenced reads were submitted to the Sequence Read Archive (NCBI) under the accession number PRJNA235907. Reads were mapped to the planarian genome using TopHat [57]; gene models were predicted using a published transcriptome [58], [59]. Differentially expressed genes were identified using the R Bioconductor package edgeR [60] with cutoffs of logCPM score ≥0 and FDR≤0.05. Changes in gene expression detected by RNA-seq were represented as linear fold changes over controls. For the differentially expressed *Schmidtea mediterranea* transcripts, we performed BLASTX against the human UniProt database (cutoff<1×10^−4^); human accession numbers were then used to assign Gene Ontology terms and perform clustering analysis using DAVID software [61], [62] with the “Panther_BP_all” and “Panther_MF_all” gene annotation settings and an Enrichment Score cutoff >1.3.

### Gene identification and cloning

For validation studies, transcript sequences were analyzed by BLASTX against protein sequences from human, mouse, fly, and nematode and identified as the top BLAST hit (Table S3). Sequences were obtained from a cDNA collection [63] or cloned into pJC53.2 [30] or pPR244 [64] using gene specific primers. GenBank accession numbers and the primers used in this study are listed in Table S3.

### Reverse transcription quantitative PCR

Total RNA was extracted and purified as described above. cDNA was synthesized using the iScript cDNA Synthesis Kit (BioRad). Reverse transcription quantitative PCR was performed on a Bio-Rad CFX Connect Real-Time System using SsoAdvanced SYBR Green Supermix (Bio-Rad) with a two-step cycling protocol and annealing/extension temperature of 58.5°C. At least three biological replicates and two technical replicates were performed for each experiment. The relative amount of each cDNA target was normalized to *Smed-β-tubulin* (accession no. DN305397). The normalized relative changes in gene expression, standard deviations, and t-tests were calculated in Bio-Rad CFX Manager Software v3.0. Primers are listed in Table S3.

### Imaging, cell counts, and statistical analysis

Images of live animals and whole mount *in situ* hybridization samples were acquired using a Leica DFC450 camera mounted on a Leica M205 stereomicroscope. Fluorescent images were acquired with a Zeiss Axio Observer.Z1 equipped with an Axiocam MRm camera and ApoTome; images are displayed as maximum image projections from ten 1-µm optical sections. For all experiments, we counted cells by hand using ImageJ Software [65], and biological replicates (n≥3) were averaged and shown as mean ± standard deviation. The number of *cintillo* ^+^, *spp-19^+^*, *spp-18^+^*, and *npl^+^* cells (Fig. 4N) was normalized to animal length (mm). We used anti-SYNAPSIN staining and *ChAT* expression to determine brain area (Fig. 6E–F), normalized to animal length (µm). To quantify *npl^+^* brain-specific neurons following amputation, *npl^+^* cells were counted in the cephalic ganglia and normalized to the average total brain area (Fig. 6G). When comparing two groups, we used a Student's t-test and significance was accepted at P<0.05.

## Supporting Information

Figure S1 *coe* is required for proper regeneration of the planarian nervous system. (**A–C**) Control and *coe(RNAi)* animals were amputated pre- and post-pharyngeally, allowed to regenerate for seven days, and the CNS morphology was analyzed in regenerating trunk fragments immunostained with anti-SYNAPSIN or anti-VC-1. Arrows in A and C denote defects in anterior commissure and photoreceptor axon patterning, respectively; arrowheads in B mark reduced anti-SYNAPSIN staining in the ventral nerve cords at the tail region (N = 10). Anterior is up. Scale bar in A = 200 µm; C = 100 µm.
(TIF)

Figure S2 The transcription factors *irx-1* and *pou4l-1* are detected in brain *ChAT^+^* neurons. Double-fluorescent *in situ* hybridization to *ChAT* and *irx-1* or *pou4l-1* (N≥3 animals). Anterior is up. Scale bar = 200 µm.
(TIF)

Figure S3 Reverse transcription quantitative PCR validation of downregulated nervous system genes in *coe(RNAi)* planarians. (**A**) RT-qPCR measuring the relative expression of selected genes following *coe* RNAi treatment. (**B**) Whole-mount *in situ* hybridization to genes that are expressed in the nervous system of planarians and did not significantly change expression levels after *coe* RNAi. The genes shown (indicated above each panel) were selected from an *in situ* hybridization screen (unpublished). The expression pattern of the neuropeptide genes *grh-1*, *ilp*, *mpl-1*, *npp-2*, *spp-15* and *spp-16* (see C) were reported in [30]. Anterior is up. (**C**) RT-qPCR measurements of gene expression for nervous system-specific genes in control and *coe* RNAi planarians. All graphs show the mean ± s.d. expression level relative to the controls; *P<0.05, Student's t-test. Scale bar in B = 200 µm.
(TIF)

Figure S4 Identification of genes expressed in *coe^+^* neurons. Additional data for Figure 5. Double-FISH to *coe* and *vamp*, *gbrg*, or *scna-1* (N≥3 animals). White arrowheads mark cells co-labeled with *coe*. Anterior is up. Scale bar = 200 µm.
(TIF)

Figure S5 *nkx2l* is required for tissue regeneration. Additional data for Figure 6. (**A**) *In situ* hybridization to *nkx2l-1*. (**B**) After 10 days of regeneration, the tail region of *control* and *nkx2l* RNAi animals were imaged live or immunostained with anti-SYNAPSIN. Anterior is up. Scale bars = 200 µm.
(TIF)

Table S1 List of differentially expressed genes following *coe* RNAi.
(XLSX)

Table S2 Expression analysis of downregulated genes following *coe* RNAi.
(XLSX)

Table S3 Accession numbers, primers, and top BLAST hits for the genes analyzed in this study.
(XLSX)
