# Supplementary figures and images for: COE Loss-of-Function Analysis Reveals a Genetic Program Underlying Maintenance and Regeneration of the Nervous System in Planarians
Source: PLoS Genet. 2014 Oct 30;10(10):e1004746. doi: 10.1371/journal.pgen.1004746 (PMC4214590; doi:10.1371/journal.pgen.1004746)

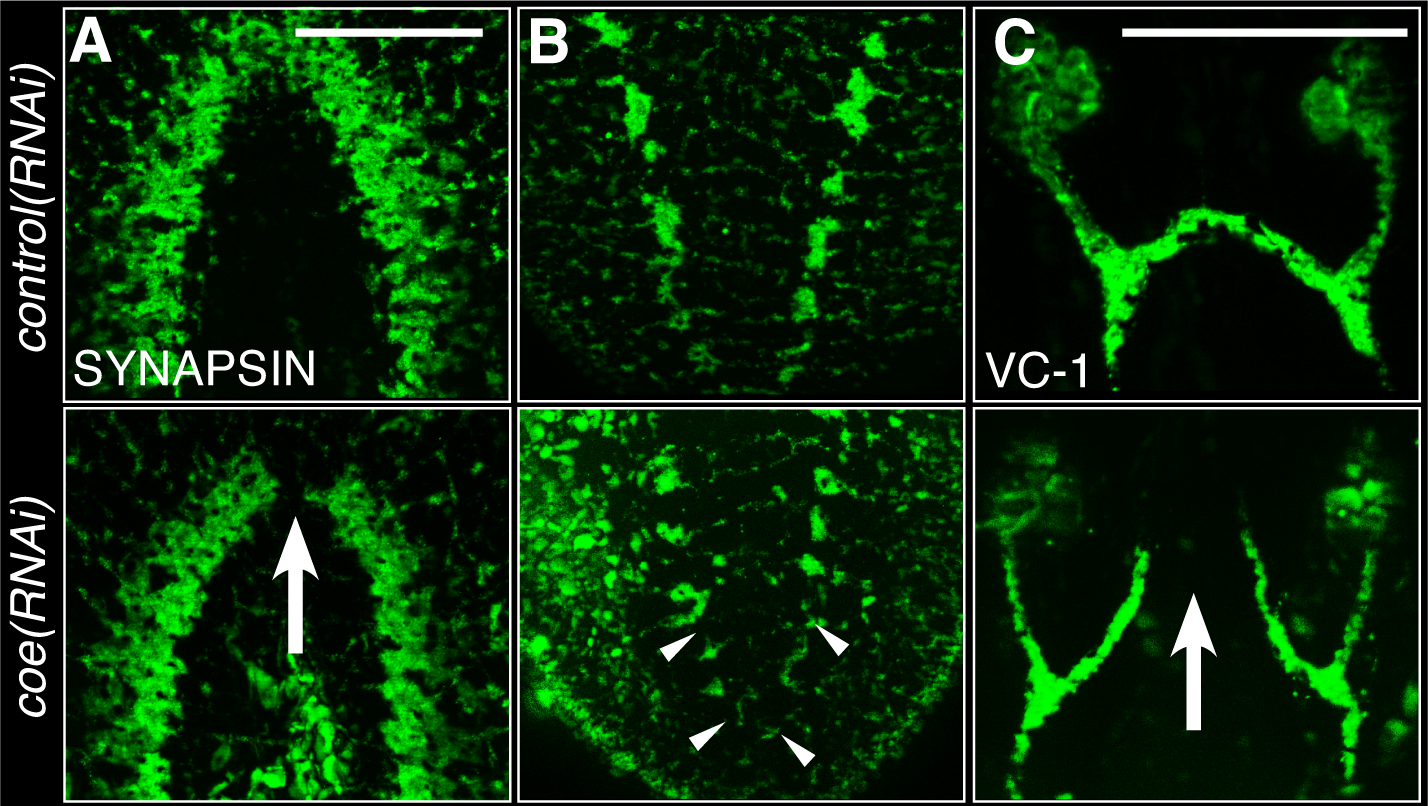

Supplement: Figure S1 — coe is required for proper regeneration of the planarian nervous system. (A–C) Control and coe(RNAi) animals were amputated pre- and post-pharyngeally, allowed to regenerate for seven days, and the CNS morphology was analyzed in regenerating trunk fragments immunostained with anti-SYNAPSIN or anti-VC-1. Arrows in A and C denote defects in anterior commissure and photoreceptor axon patterning, respectively; arrowheads in B mark reduced anti-SYNAPSIN staining in the ventral nerve cords at the tail region (N = 10). Anterior is up. Scale bar in A = 200 µm; C = 100 µm. (TIF) [file pgen.1004746.s001.tif]

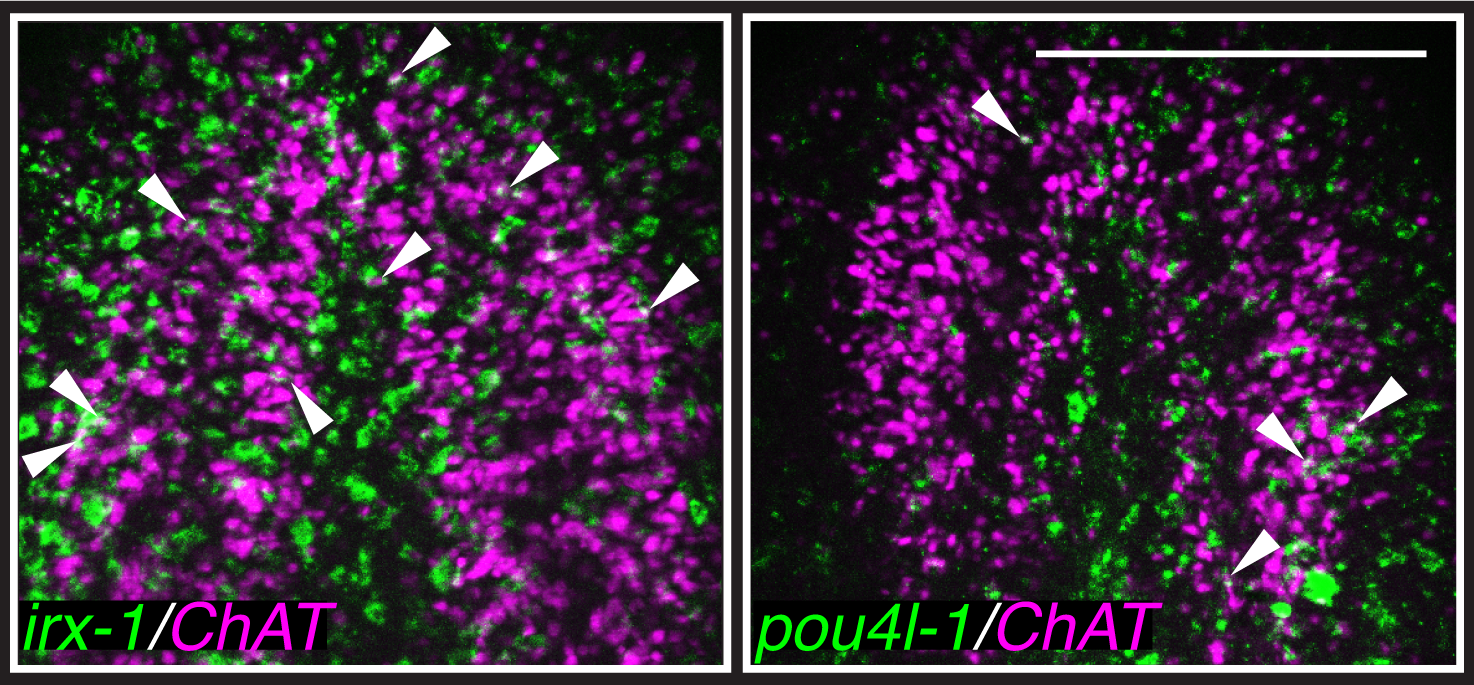

Supplement: Figure S2 — The transcription factors irx-1 and pou4l-1 are detected in brain ChAT+ neurons. Double-fluorescent in situ hybridization to ChAT and irx-1 or pou4l-1 (N≥3 animals). Anterior is up. Scale bar = 200 µm. (TIF) [file pgen.1004746.s002.tif]

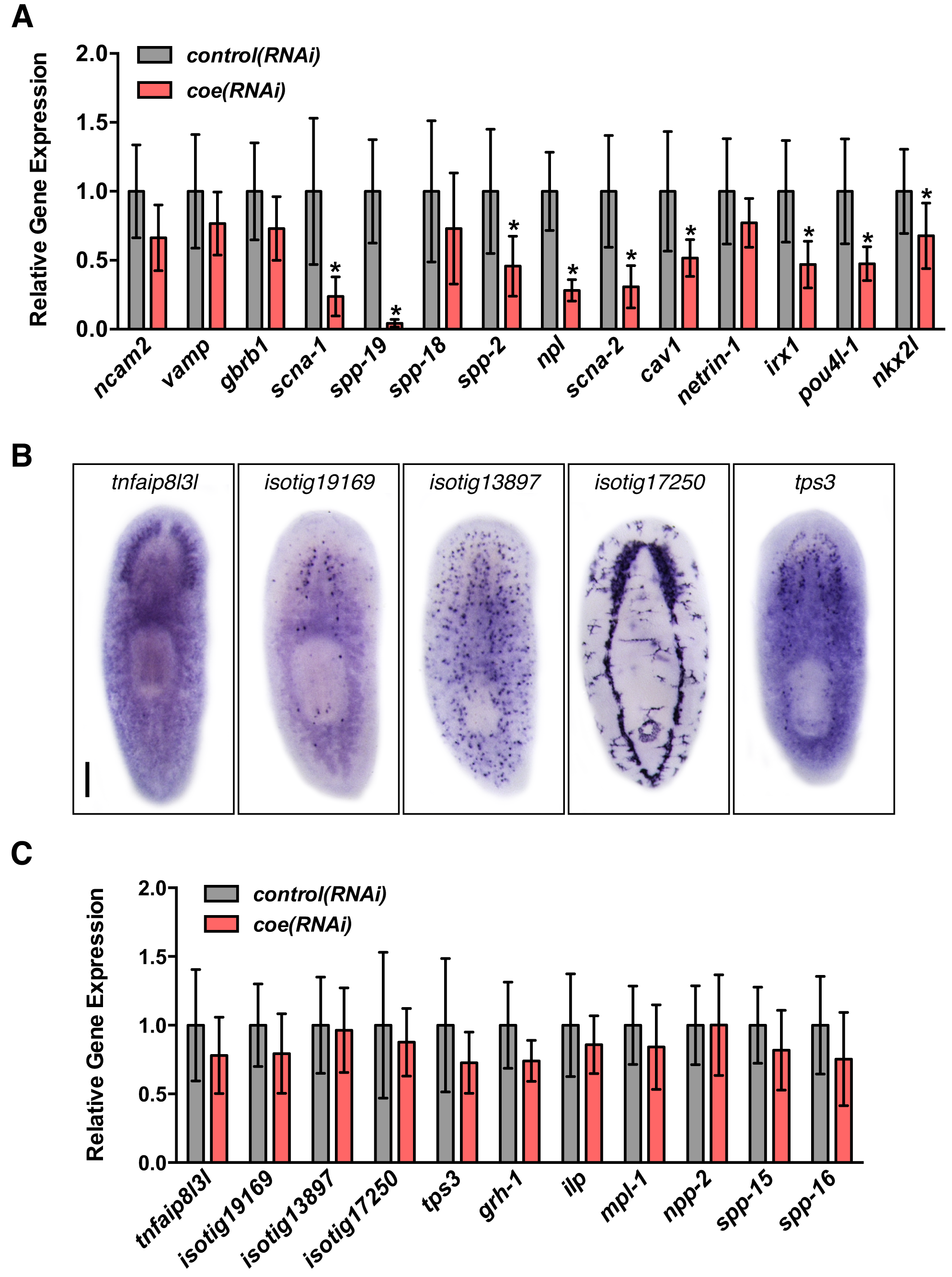

Supplement: Figure S3 — Reverse transcription quantitative PCR validation of downregulated nervous system genes in coe(RNAi) planarians. (A) RT-qPCR measuring the relative expression of selected genes following coe RNAi treatment. (B) Whole-mount in situ hybridization to genes that are expressed in the nervous system of planarians and did not significantly change expression levels after coe RNAi. The genes shown (indicated above each panel) were selected from an in situ hybridization screen (unpublished). The expression pattern of the neuropeptide genes grh-1, ilp, mpl-1, npp-2, spp-15 and spp-16 (see C) were reported in [30]. Anterior is up. (C) RT-qPCR measurements of gene expression for nervous system-specific genes in control and coe RNAi planarians. All graphs show the mean ± s.d. expression level relative to the controls; *P<0.05, Student's t-test. Scale bar in B = 200 µm. (TIF) [file pgen.1004746.s003.tif]

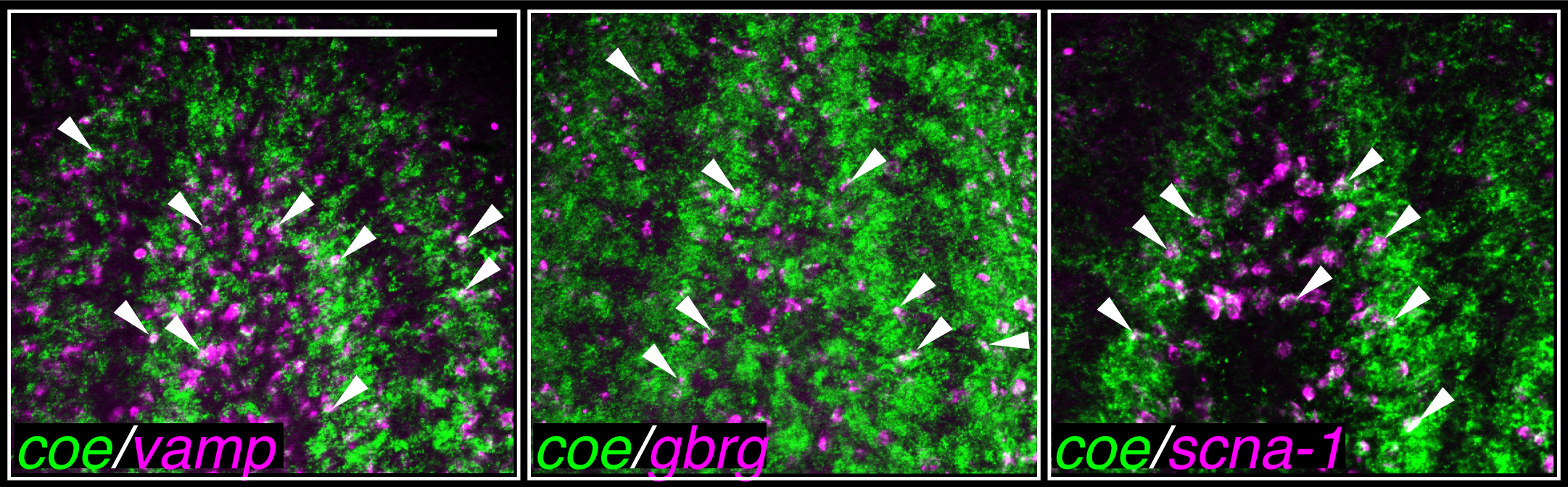

Supplement: Figure S4 — Identification of genes expressed in coe+ neurons. Additional data for Figure 5. Double-FISH to coe and vamp, gbrg, or scna-1 (N≥3 animals). White arrowheads mark cells co-labeled with coe. Anterior is up. Scale bar = 200 µm. (TIF) [file pgen.1004746.s004.tif]

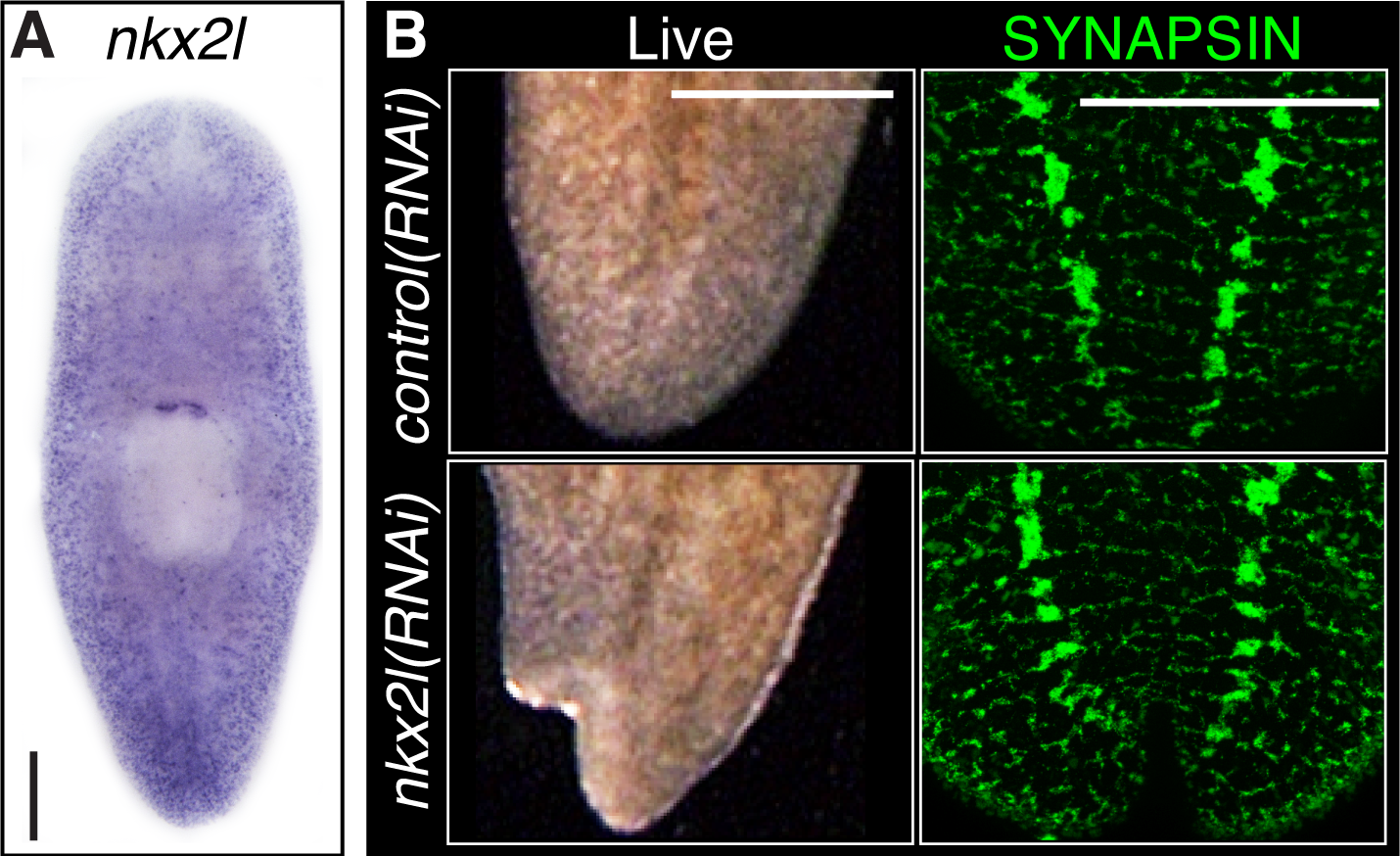

Supplement: Figure S5 — nkx2l is required for tissue regeneration. Additional data for Figure 6. (A) In situ hybridization to nkx2l-1. (B) After 10 days of regeneration, the tail region of control and nkx2l RNAi animals were imaged live or immunostained with anti-SYNAPSIN. Anterior is up. Scale bars = 200 µm. (TIF) [file pgen.1004746.s005.tif]
